# Grape Berry Secondary Metabolites and Their Modulation by Abiotic Factors in a Climate Change Scenario–A Review

**DOI:** 10.3389/fpls.2021.643258

**Published:** 2021-03-22

**Authors:** Markus Rienth, Nicolas Vigneron, Philippe Darriet, Crystal Sweetman, Crista Burbidge, Claudio Bonghi, Robert Peter Walker, Franco Famiani, Simone Diego Castellarin

**Affiliations:** ^1^Changins College for Viticulture and Oenology, University of Sciences and Art Western Switzerland, Nyon, Switzerland; ^2^Unité de recherche Œnologie EA 4577, USC 1366 INRAE, Bordeaux, France; ^3^Institut des Sciences de la Vigne et du Vin CS 50008, Villenave d'Ornon, France; ^4^College of Science & Engineering, Flinders University, Bedford Park, SA, Australia; ^5^Agriculture and Food (Commonwealth Scientific and Industrial Research Organisation), Glen Osmond, SA, Australia; ^6^Department of Agronomy, Food, Natural Resources, Animals and Environment, University of Padova Agripolis, Legnaro, Italy; ^7^Dipartimento di Scienze Agrarie, Alimentari e Ambientali, Università degli Studi di Perugia, Perugia, Italy; ^8^Faculty of Land and Food Systems, Wine Research Centre, The University of British Columbia, Vancouver, BC, Canada

**Keywords:** grapevine berry, climate change, abiotic stress, secondary metabolism, phenolic compounds, aroma compounds, *Vitis vinifera*

## Abstract

Temperature, water, solar radiation, and atmospheric CO_2_ concentration are the main abiotic factors that are changing in the course of global warming. These abiotic factors govern the synthesis and degradation of primary (sugars, amino acids, organic acids, etc.) and secondary (phenolic and volatile flavor compounds and their precursors) metabolites directly, via the regulation of their biosynthetic pathways, or indirectly, via their effects on vine physiology and phenology. Several hundred secondary metabolites have been identified in the grape berry. Their biosynthesis and degradation have been characterized and have been shown to occur during different developmental stages of the berry. The understanding of how the different abiotic factors modulate secondary metabolism and thus berry quality is of crucial importance for breeders and growers to develop plant material and viticultural practices to maintain high-quality fruit and wine production in the context of global warming. Here, we review the main secondary metabolites of the grape berry, their biosynthesis, and how their accumulation and degradation is influenced by abiotic factors. The first part of the review provides an update on structure, biosynthesis, and degradation of phenolic compounds (flavonoids and non-flavonoids) and major aroma compounds (terpenes, thiols, methoxypyrazines, and C13 norisoprenoids). The second part gives an update on the influence of abiotic factors, such as water availability, temperature, radiation, and CO_2_ concentration, on berry secondary metabolism. At the end of the paper, we raise some critical questions regarding intracluster berry heterogeneity and dilution effects and how the sampling strategy can impact the outcome of studies on the grapevine berry response to abiotic factors.

## Grape Berry Phenolics

Phenolic compounds constitute a large group of secondary metabolites, which are produced via different branches of the phenylpropanoid pathway. They are widely distributed throughout the plant kingdom and function as pigments, antioxidants, signaling molecules, structural elements, and components of defense mechanisms (Rienth et al., 2019; Santos-Sánchez et al., 2019).

**Figure 1:**
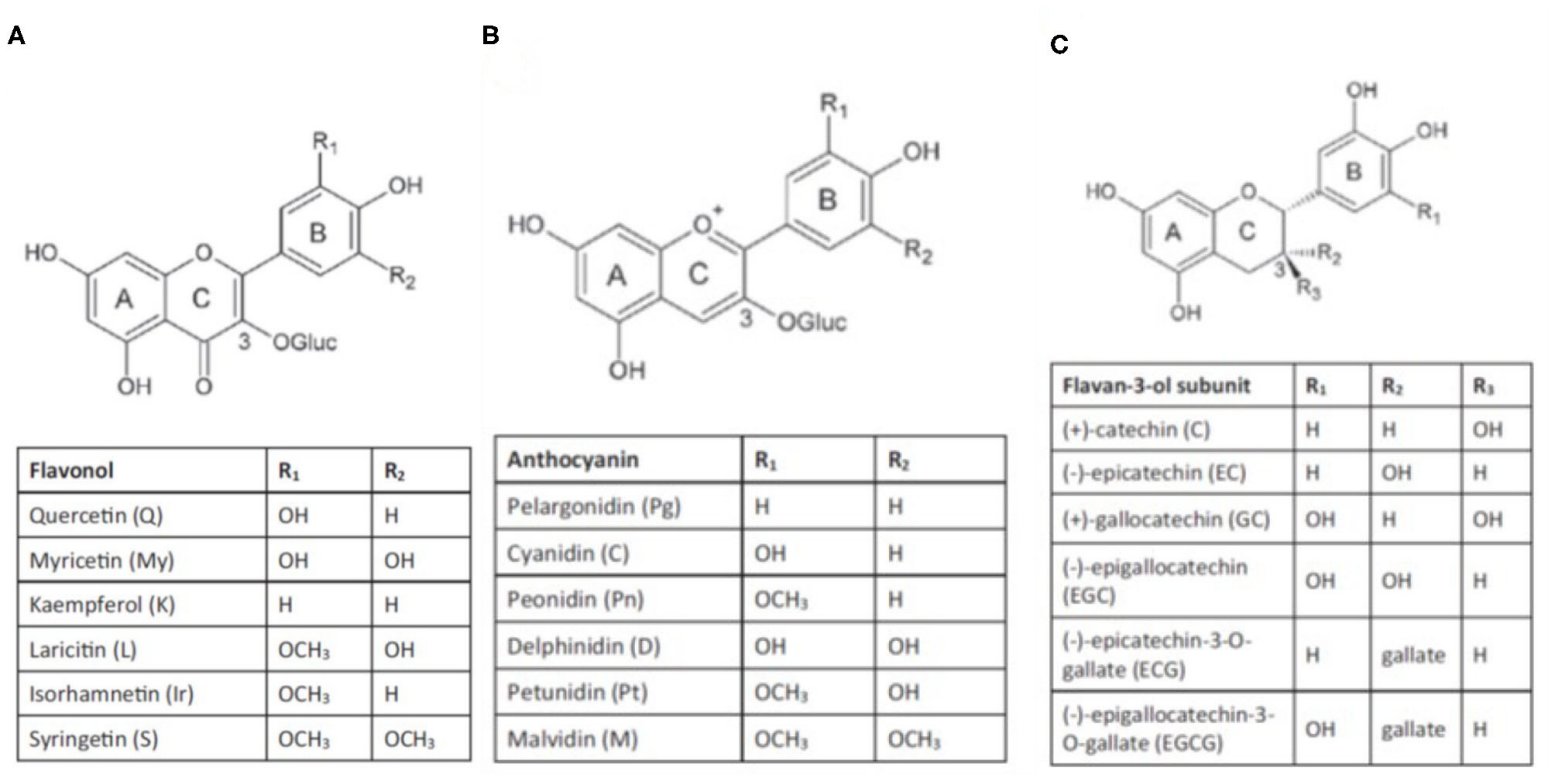
Structure of flavonols **(A)**, anthocyanins **(B)** and flavan-3-ols **(C)** (from Gouot et al., 2018).

They are composed of a phenyl ring backbone with a hydroxyl group or other substitutes and are generally classed into non-flavonoids and flavonoids. Non-flavonoids consist of simple C6 backbone phenolics such as hydroxybenzoic acids, hydroxycinnamic acids, and volatile phenols, and C6-C2-C6 backbone compounds such as stilbenes. Flavonoid compounds that occur in grapes comprise flavones, flavonols, flavanones, flavan-3-ols, and anthocyanins (Figure 1).

Phenolic compounds are synthesized via the general phenylpropanoid pathway (GPP) and its downstream reactions. After the generation of intermediates in the GPP, the carbon flow is directed into specific branch pathways to produce flavonoids, stilbenes, and phenolic acids (Deng and Lu, 2017). The starting point of the GPP is the aromatic amino acid phenylalanine, a product of the shikimate pathway, which constitutes a major link between primary and secondary metabolism in vascular plants (Santos-Sánchez et al., 2019).

The GPP leads to the production of p-coumaroyl-CoA from phenylalanine, via reactions catalyzed by phenylalanine ammonia lyase (PAL), cinnamate-4-hydroxylase (C4H), and 4-coumarate-CoA ligase (4CL). Further downstream p-coumaroyl-CoA and malonyl-CoA are catalyzed by chalcone synthase (CHS) and stilbene synthase (STS) in the first steps of the flavonoid and stilbenoid pathway, respectively.

Recently, two transcription factors (TFs), *VviMYB4a* and *VviMYB4b*, have been characterized as negative regulators of phenylpropanoid and hydrocinnamic acid synthesis (Cavallini et al., 2015) and can repress upstream genes of the phenylpropanoid biosynthesis pathway in berry skins (Muñoz et al., 2019).

### Non-flavonoid Phenolics

The concentrations of non-flavonoid phenolic compounds present in grapes and wines are relatively low (25–60 mg L^−1^), with the exception of hydroxycinnamic acids (150–200 mg L^−1^) (Kennedy et al., 2006). They are principally located in the berry pulp and are the major phenolic compounds in berries of white cultivars, although they reach similar magnitudes in reds (Kennedy et al., 2006).

The major non-flavonoid compounds present in grapes are the hydroxycinnamic acids: p-coumaric acid, caffeic acid, ferulic acid, and their esterified forms, coutaric, caftaric, and fertaric acid (Zhang et al., 2013). Their biosynthesis occurs during the first phase of berry growth until the lag phase (herbaceous plateau) and is catalyzed by caffeic acid 3-O-metyltransferase (COMT) and caffeoyl-CoA 3-O-methyltransferase (CCoAOMT) downstream of the GPP. Although the accumulation occurs predominantly in the flesh, they are present in all berry tissues (Cadot et al., 2006; Braidot et al., 2008). In hypodermal, mesocarp, and placental cells of the pulp, hydroxycinnamates may be conjugated with anthocyanins (Cadot et al., 2006; Conde et al., 2007; Castellarin et al., 2012; Kuhn et al., 2013). Minor non-flavonoid compounds comprise hydroxybenzoic acids that are present in grapes in much lower concentrations than hydroxycinnamic acids, and include gentisic acid, salicylic acid, gallic acid, and p-hydroxybenzoic acid (Vanhoenacker et al., 2001; Pozo-Bayon et al., 2003; Ali et al., 2010). The most represented hydroxybenzoic acid is gallic acid, which is present in both free form or, in the seed, as an acyl substituent of flavan-3-ols (described below) (Adams, 2006).

Grape stilbenoids include *cis*- and *trans*-resveratrol, piceatannol, *cis*- and *trans*-piceid, astringin, pallidol and α-, β-, γ-, δ-, ε-viniferin. Although stilbenoids are present in trace quantities in wine, they have been drawing increasing attention due to their potential health benefits (Akinwumi et al., 2018). They have been shown to protect the plant against various pathogens and have been detected in significant concentrations in leaves and berries of highly stressed vines and disease-tolerant *Vitis* species such *V. amurensis* (Kiselev et al., 2017). Therefore, they represent important biomarkers and a molecular target for breeding strategies aiming to develop disease-resistant cultivars (Gindro et al., 2012; Viret et al., 2018). Recent research seeking alternatives to pesticides highlighted the potential of stilbenes extracted from wooden grapevine tissue as natural fungitoxic substances (Schnee et al., 2008; Biais et al., 2017).

Stilbenes are mostly accumulated in berries after the onset of ripening (*véraison*) (Gatto et al., 2008), and the regulation of their biosynthesis is strongly modulated by both biotic and abiotic factors (Vannozzi et al., 2012; Savoi et al., 2017). The concentration of stilbenes in grapes is variety-dependent; generally, red grapes have higher stilbene levels than white grapes (Bavaresco et al., 2016). Reported levels in grapes range from 0.2 to 1.8 mg kg^−1^ fresh weight of healthy grapes for cultivars that were classified as low stilbene producers (e.g., Nebbiolo or Aglianico) but can reach levels up to 33.4 mg kg-1 in high stilbene producers (> 2.3 mg kg^−1^) such as Pinot Noir (Gatto et al., 2008) or Syrah (Favre et al., 2020).

Stilbenes can be glycosylated or methylated. The stilbene with the simplest molecular structure is resveratrol, which exists as a *trans* or *cis* isomer (Kiselev et al., 2017). Resveratrol has been associated with the so called “French paradox,” where the rather low risk of cardiovascular disease within the French population despite of a high intake of saturated fat was attributed to relatively high resveratrol intake through wine consumption (Renaud and de Lorgeril, 1992). In subsequent studies, resveratrol was designated the main substance amongst other phenolic compounds accountable for such protective effects (Pastor et al., 2019). Its anticancer, anti-inflammatory, anticarcinogenic, cardioprotective, vasorelaxant, phytoestrogenic, and neuroprotective properties have been reported in numerous studies (Guerrero et al., 2009; Chalons et al., 2018; Ramírez-Garza et al., 2018; Salehi et al., 2018; Wurz, 2019).

Resveratrol is the precursor for other stilbenoids such as piceids, *trans*- and *cis*-resveratrol-3-O-β-D-glucopyranosidade, and astringin. Pterostilbene (3,5-dimethoxy-4′-hydroxystilbene), which exhibits an enhanced antifungal activity compared to the non-methylated stilbenoid forms, is formed by the addition of two methyl groups to resveratrol (Chong et al., 2009). The oxidation of resveratrol produces oligomers called viniferins. The most important viniferins are α*-*, β*-*, γ*-*, δ*-*, and ε-viniferin, consisting essentially of cyclic oligomers of resveratrol (Castellarin et al., 2012).

In the grapevine genome, forty-five stilbene synthases genes (*STS*), the key enzyme of resveratrol biosynthesis, have been described, of which at least 33 encode full-length proteins. Multiple tandem and segmental duplication events led to the rise of this gene family (Vannozzi et al., 2012). Many of those *VviSTSs* exhibit changing expression pattern during fruit development and ripening as shown by transcriptomic analysis (Massonnet et al., 2017). In red berry cultivars, the expression of *VviSTSs* is increased during the late stages of ripening, consistently with the expression of two *R2R3 MYB* TFs, *VviMYB14* and *VviMYB15* (Holl et al., 2013), which are known to regulate stilbene biosynthesis. Amongst the many TFs proposed to regulate this pathway (Wong et al., 2016; Vannozzi et al., 2018) the two WRKY TFs, *VviWRKY24* and *VviWRKY03*, contribute at different levels to *VviSTS* regulation. This occurs by a direct activation of *VviSTSs* and synergistic action with MYB TFs. Recently, Jiang et al. (2019) indicated that *VvWRKY8* represses *VvSTS15/21* expression and thus stilbene biosynthesis through the interaction with *VvMYB14*.

### Flavonoid Phenolics

**Flavonoids** make up a significant proportion of phenolic compounds in red grapes (1,000–1,800 mg L^−1^) and can be considered as the most important quality-determining compounds in red wines as they contribute to color, flavor, texture, and astringency. Flavonoids are C6–C3–C6 polyphenolic compounds, where the two hydroxylated benzene rings are joined by a three-carbon chain. According to the oxidation state of the C3 ring, these compounds are divided into flavonols, flavan-3-ols (which include simple flavan-3-ols and their polymeric forms proanthocyanidins, also known as tannins), and anthocyanins (Castellarin et al., 2012). Flavonoids are mainly localized in both the peripheral layers of the berry pericarp (skin) and in the seed coat (Teixeira et al., 2013).

The flavonoid pathway has been well-characterized in grapevine. Most of the genes encoding structural elements of the flavonoid pathway are present in low copy numbers except genes coding *flavonoid-3*′*,5*′*-hydroxylases* (*F3*′*5*′*H*′s). p-Coumaroyl-CoA and malonyl-CoA are the substrates of *chalcone synthase (CHS)*, which catalyzes the first step of flavonoid synthesis. Subsequently, naringenin chalcone is transformed to naringenin flavanone by *chalcone isomerase (CHI)* and to dihydrokaempferol by *flavonoid-3-hydrolase (F3H)*. The pathway is then divided into two major branches catalyzed by two flavonoid hydroxylases, *flavonoid-3*′*-hydroxylase (F3*′*H)* and *flavonoid-3*′*5*′*-hydroxylase (F3*′*5*′*H)*, which generate di-hydroxylated and tri-hydroxylated flavonoids, respectively (Azuma, 2018). In the grapevine genome a proliferation of the *F3*′*5*′*H*s has occurred, giving rise to 15 paralogs that are predominantly expressed in grapes (Falginella et al., 2010, 2012).

**Flavonols**, such as kaempferol, quercetin, myricetin, isorhamnetin, laricitrin, and syringetin are synthesized by *flavonol synthases (FLSs)* (Downey et al., 2004), regulated by a light-induced TF (*VviMYBF1/VviMYB12*) (Czemmel et al., 2009). Two studies showed that three additional bZIP TFs, *VviHYH, VviHY5*, and *VvibZIPC22*, contribute to the regulation of flavonol synthases and the accumulation of flavonol in berries (Malacarne et al., 2015; Loyola et al., 2016). The TF *VviMYBF1* is part of a regulatory cascade of *VviHY5/HYH* that potentially involves positive feedback (Loyola et al., 2016; Czemmel et al., 2017). Flavonols are glycosylated by *flavonol-3-O-glycosyltransferases (VviGT3-5-6)* and *flavonol-3-O-rhamnosyltransferase (VviRhaT1)* and are present in the berry as galactosides, rhamnosides, rutinosides, and glucuronides (Ono et al., 2010; Czemmel et al., 2017).

**Flavan-3-ols** are synthesized from anthesis until the lag phase via *leucoanthocyanidin reductases (LAR1-2)* or an *anthocyanidin reductase (ANR)* (Bogs et al., 2005), which are regulated by TFs of the MYB family. In particular, *VviLAR1* and *VviANR* are under the control of *VviMYBPA1* and *VviMYBPA2* (Bogs et al., 2007; Terrier et al., 2009), whereas *VviLAR2* is regulated by *VviMYBPAR* (Koyama et al., 2014). In grapes, the main monomeric flavan-3-ols are (+)-catechin, (-)-epicatechin, (-)-epicatechin-3-O-gallate, (+)-gallocatechin and (-)-epigallocatechin (Mattivi et al., 2009), and are the building blocks for pro-anthocyanidins (condensed tannins). The mechanisms involved in tannin polymerization, galloylation, and transport into the vacuoles are not well-elucidated (Zhao et al., 2010). Three different glycosyltransferases (*VviGT1-3*) are ostensibly involved in the synthesis of hydroxycinnamic esters and in the galloylation of proanthocyanidins (Khater et al., 2012), and two specific transporters of proanthocyanidin have been identified (*VviPAMATE1-2)* (Perez-Diaz et al., 2014).

**Anthocyanins** are the pigments that are responsible for the color of red grapes and wines. They are synthesized in the epidermal and hypodermal cells of the berry skin and stored in the cell vacuoles from the onset of ripening (*véraison*). Few red cultivars, commonly called by the French word “Teinturier”, accumulate anthocyanins also in the flesh (Ageorges et al., 2006; Castellarin et al., 2011; Falginella et al., 2012).

In *Vitis vinifera*, anthocyanins are glycosylated at the 3′ position. They can be substituted with two (di-oxygenated: cyanidin- and peonidin-3-O-glucosides) or three (tri-oxygenated: delphinidin-, petunidin-, and malvidin-3-O-glucosides) hydroxyl (-OH) and/or methoxyl (-OCH3) groups in the side-ring (B) of the flavonoid structure. Glycosylation occurs via the activity of the enzyme UDP-glucose, flavonoid-3-O-glucosyltransferase (UFGT) (Boss et al., 1996a,b). Anthocyanin-O-methyl transferases (*VviAOMT1-3*) methylate cyanidin-3-O-glucoside and delphinidin-3-O-glucoside into peonidin-3-O-glucoside, petunidin-3-O-glucoside, and malvidin-3-O-glucoside (Fournier-Level et al., 2011). Most anthocyanins can be acylated at the 6′ position of the glucose mediated by an anthocyanin-3-O-glucoside-6′-O-acyltransferase (*Vvi3AT*) to produce 3-O-6′-acetyl-, 3-O-6′-coumaroyl- and 3-O-6′-caffeoyl-monoglucosides (Rinaldo et al., 2015). Anthocyanins composition (the relative portion of individual anthocyanins, the ratio of di-oxygenated vs. tri-oxygenated side-ring forms, the ratio of acylated vs. non-acylated derivatives, etc.) is variable among grapevine cultivars (Monagas et al., 2003; Theodorou et al., 2019) and can be modified by abiotic factors as discussed in later sections.

The two TFs *VviMYBA1* and *A2* are the pivotal genetic determinants of berry anthocyanin synthesis (Kobayashi et al., 2004; Walker et al., 2007). Recently it has been shown that *VviMYBA6* and *VviMYBA7*, additional MYBA family members, regulate anthocyanin biosynthesis in vegetative organs (Matus et al., 2017) and that both also possess the capacity to alter fruit anthocyanin pigmentation and composition under extreme abiotic conditions (i.e., UV-B) during ripening (Czemmel et al., 2017). Recently, Costantini et al. (2017) identified a set of new candidate genes responsible for anthocyanin variation among cultivars.

The translocation of anthocyanin-acylglucosides into the vacuole is mediated by MATE-type transporters localized in the tonoplast (*VviAnthoMATE1-3*) (Gomez et al., 2009), whereas translocation of glycosylated anthocyanins occurs via a glutathione- dependent, ATP-binding cassette (ABC) protein (*VviABCC1*) (Francisco et al., 2013). Furthermore, glutathione S-transferases (*VviGST*s) have been associated with the transport of anthocyanins (Conn et al., 2008). Notably, *VviGST1* and *VviGST4* have been shown to be involved in anthocyanin accumulation in the vacuole (Pérez-Díaz et al., 2016).

The synthesis of hydroxycinnamic acids, stilbenes, flavonols, flavan-3-ols, and anthocyanins is spatially and temporally separated during grape berry development and tightly regulated by a vast transcriptional gene network. In addition to the already described TFs, two MYB (*VviMYB5a-b*) have been shown to be general regulators of the flavonoid pathway and, in particular, modulate the expression profile of several flavonoid genes such as *VviCHI, VviF3*′*5*′*H, VviLDOX, VviLAR*, and *VviANR* during berry development (Deluc et al., 2006; Cavallini et al., 2015). Recently, several R2R3-MYBs (*VviMyb4a, VviMyb4b, VviMybC2-L1, VviMybC2-L2, VviMybC2-L3*, and *VviMyb4-like*) were characterized as repressors of both proanthocyanidin and anthocyanin biosynthesis (Huang et al., 2014; Cavallini et al., 2015; Muñoz et al., 2019). Moreover, a bHLH (*VviMYC1*) interacts with *VviMYB5a-b, VviMYBPA1*, and *VviMYBA1-A2* in the transcriptional regulation of proanthocyanidin and anthocyanins biosynthesis in grapevine (Hichri et al., 2011).

## Grape Berry Aroma Compounds

In this section, a brief overview of aroma compound formation in grape berries is given to lay the basis for subsequent parts on abiotic interaction with their synthesis. For more detailed information we refer the reader to the recently published review of Lin et al. (2019).

The major groups of grape and wine aroma compounds are mono- and sesquiterpenes, methoxypyrazines, furan derivatives, lipoxygenase pathway products, phenylpropanoid pathway products, norisoprenoids, and volatile sulfur compounds such as thiols. They are mainly found as non-volatile precursors in the berry and require further modifications to be perceived (Dunlevy et al., 2013; Parker et al., 2018; Lin et al., 2019).

### Terpenes

Terpenes play roles as phytohormones, protein modification reagents, anti-oxidants, repellents of herbivores and attractants of predators and parasitoids of herbivores (Pichersky and Raguso, 2018). The grape terpenoids of major aromatic importance in grapes can be divided according to their chemical structure into monoterpenes (C10) and sesquiterpenes (C15). Moreover, carotenoids (C40) can be included in this list as aroma precursors (Dunlevy et al., 2013; Ilc et al., 2016).

**Figure 2:**
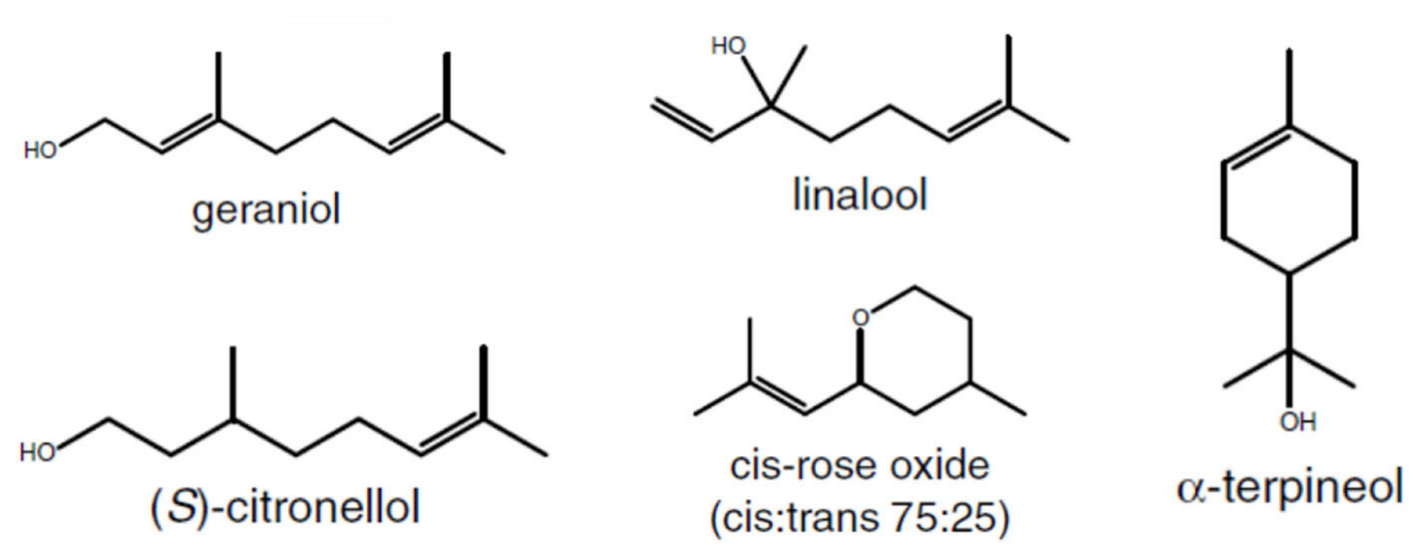
Structure of main monoterpenes found in grapes.

Around 50 monoterpenes (including *cis* and *trans* forms) have been identified in grapes and wine with a significant proportion of them being linalool derivatives (Strauss et al., 1986; Black et al., 2015; Ilc et al., 2016). The main monoterpenes present in grapes are linalool, geraniol, nerol, citronellol, *(E)*-hotrienol, α-terpineol, and rose oxides (Figure 2) (Matarese et al., 2014), which confer flowery and fruity notes to wines (Siebert et al., 2018). Generally, in aromatic cultivars, terpene concentrations peak early during green berry development, decline until *véraison*, and strongly increase during ripening (De Billerbeck et al., 2003; Matarese et al., 2013; Costantini et al., 2017). Inversely, Poitou et al. (2017b) found 1,8-cineole concentration to decrease during ripening. 1,8-cineole, commonly known as eucalyptol, is the major aroma compound present in the leaves of many *Eucalyptus* species. In Australian wines, the presence of this compound has been attributed to the proximity of vineyards to eucalyptus trees and the incorporation of *Eucalyptus* material into harvested grapes (Capone et al., 2012). 1,8-cineole was also identified as a contributor to the varietal aroma reminiscent of menthol and overall green perception of unripe grapes of the cultivars from the Carmenet family, and partially linked to the proximity of the invasive plant *Artemisia verlotiorum* (Poitou et al., 2017b).

Grape cultivars can be grouped roughly into three classes based on their total free monoterpene concentration and their monoterpene profile in their wines: cultivars named “neutral” (e.g., Chardonnay and Chasselas) have low terpene concentrations, cultivars more “aromatic” (e.g., Gewürztraminer, Albariño, Scheurebe, Auxerrois, and Riesling) have moderate (1–4 mg L^−1^) terpene concentrations, and “Muscat type” cultivars (e.g., Muscat de Frontignan, Muscat of Alexandria, Muscat d'Ottonel, Muscat de Hambourg, White Muscat, etc.) have high (as much as 6 mg L^−1^) terpene concentrations (Darriet et al., 2012).

Sesquiterpenes contribute little to grape and wine aroma, as their concentrations are often below the olfactory threshold. The most studied sesquiterpene is (-)-rotundone, which confers a peppery character to some red and white cultivars, such as Syrah, Duras, Durif, and Viognier (Wood et al., 2008; Geffroy et al., 2014, 2018, 2019).

Terpenes are produced via two independent pathways: (1) the plastidial 2C-methyl-D-erythritol-4-phosphate (MEP) pathway, which is the predominant pathway for monoterpenes (C10) and diterpenes (C20), and (2) the cytosolic mevalonate (MVA) pathway, which is the primary pathway for sesquiterpenes (Bohlmann and Keeling, 2008).

The key enzyme of the MEP pathway in grapevine has been shown to be 1-deoxy-D-xylulose 5-phosphate synthase (*VviDXS*) (Battilana et al., 2009, 2011; Duchene et al., 2009a). Further downstream terpene synthases (TPSs) regulate the monoterpene or sesquiterpene production (Martin et al., 2010; Matarese et al., 2013, 2014). The genome of *Vitis vinifera* contains 69 putative TPSs, of which 39 were functionally characterized (Martin et al., 2010) and can be divided into seven clades: TPS-a, -b, -c, -d, -e/f, -g, and -h (Chen et al., 2011). The TPS-a clade (30 genes) is mainly composed of sesquiterpene and possibly diterpene synthases, whereas the TPS-b clade (19 genes) and TPS-g clade (17 genes) is composed principally of monoterpene synthases and the TPS-c (2 genes) and TPS-e/f (1 gene) clades is composed of genes related to plant hormone metabolism and are typically present in a single copy in plant genomes. No full-length TPS-d and TPS-h are known yet in grapevine (Martin et al., 2010). Several genes, such as nudix hydroxylase, vesicle-associated proteins, ABCG transporters, glutathione S-transferases, and amino acid permeases are potential candidate genes for monoterpene biosynthesis regulation and accumulation in the berry (Costantini et al., 2017). Cramer et al. (2014) hypothesized that ethylene signaling could play a role in the biosynthesis of terpenes which has been point out by a positive correlation between aroma production and ERF TFs. The hormones jasmonic acid and methyljasmonate are putatively involved as major regulators of terpene biosynthesis in grapes (Savoi et al., 2016; D'Onofrio et al., 2018). The key genes involved in the synthesis of the sesquiterpene (-)-rotundone have been identified as *VviGuaS, VviTPS24*, and *VviSTO2* (Takase et al., 2015; Drew et al., 2016).

Most monoterpenes and sesquiterpenes are present in berries as non-volatile terpene glycosides. So far, only three monoterpenol glycosyltransferases have been characterized in grapevine, *VviGT7-14-15* (Li et al., 2017). Also, the cytochrome P450 *CYP76F14* is implicated in several enzymatic transformations of monoterpenes including hydroxylation (mono- and poly- hydroxylations), cyclization, and oxidation through successive reactions including dehydration of alcohol functions in acidic media. These phenomena can lead to an increase in the diversity of monoterpenes in grapes and wines (Schwab and Wüst, 2015). As an example, cytochrome P450 catalyzes the conversion of linalool to *(E)*-8-carboxylinalool, which, during wine fermentation, generates a wine-lactone, a key odorant of Gewürztraminer wines (Ilc et al., 2016).

### Norisoprenoids

**Figure 3:**
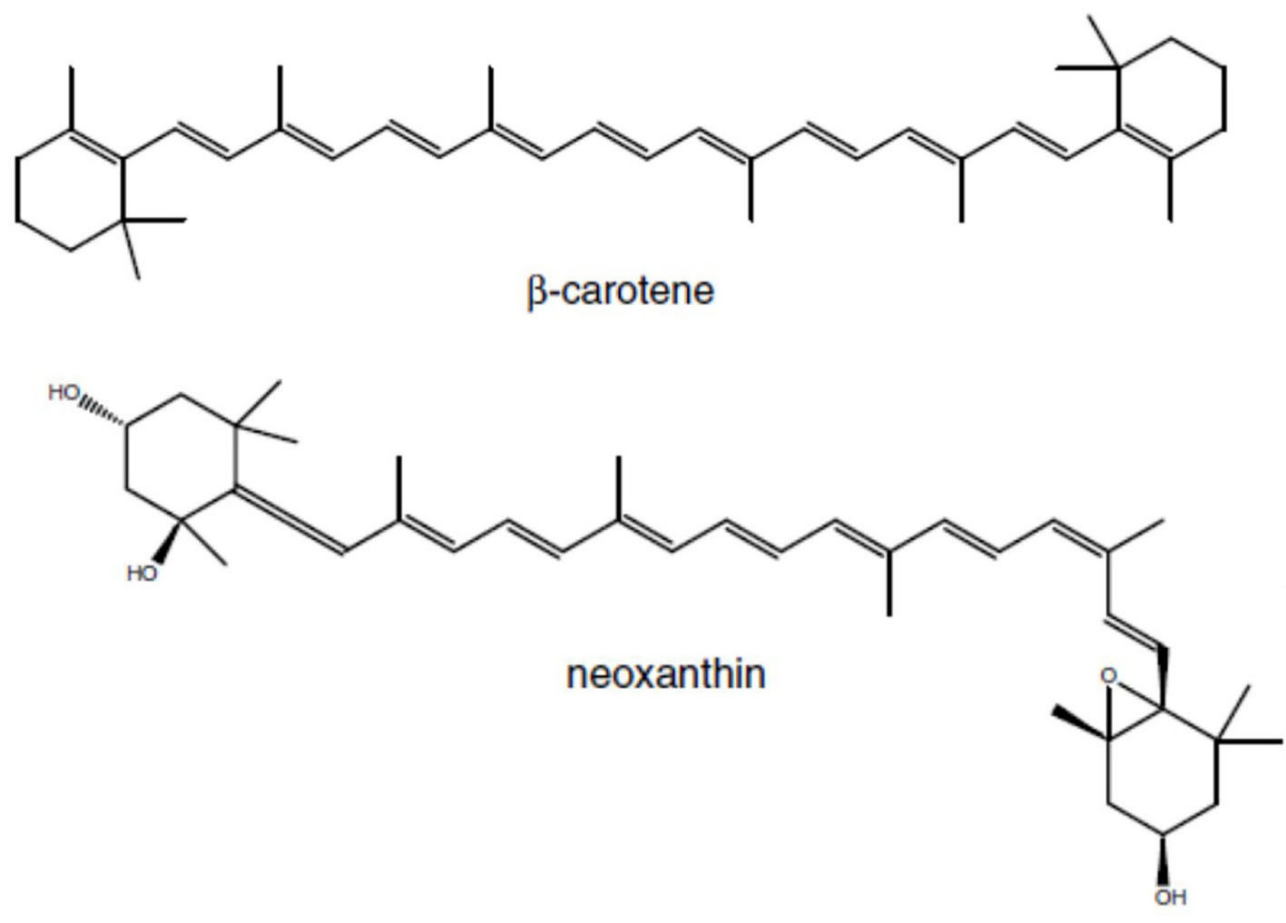
Examples of carotenoid precurors for C13 Norisprenoids in grapes: β-carotene is the precureur of β-ionone, and neoxanthin the precurseur of β-damascenone.

Norisoprenoids are a diverse group of widespread compounds derived from the oxidative breakdown of carotenoids (Figure 3) – pigments that contribute to light harvesting and to the protection of the photosynthetic apparatus from photooxidation (Rodríguez-Concepción et al., 2001). Carotenoids are synthesized from isopentenyl diphosphate (IPP) and its double-bond isomer dimethylallyl diphosphate (DMAPP) via the MEP pathway with DXS as the rate-limiting enzyme.

**Figure 4:**
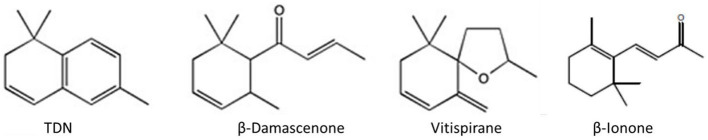
Main C13 norisporenoids in grapes.

In grapevine, carotenoids are cleaved via carotenoid cleavage dioxygenases (CDD) to form norisoprenoids with C13 carbons (C13-norisoprenoids) such as β-ionone, β-damascenone, vitispirane, actinidol, 4-(2,3,6-trimethylphenyl) buta-1,3-diene (TPB), 1,1,6-trimethyl-1,2-dihydronaphthalene (TDN), and 2,2,6-trimethylcyclohexanone (TCH) (Figure 4). Odorous C10 norisoprenoids such as safranal and ß-cyclocytral have also been found in several grape cultivars (Poitou et al., 2017a).

The cleavage of carotenoids occurs after *véraison*, during berry ripening, where a simultaneous increase in C13-norisoprenoid content correlates with a decrease in carotenoid content (Razungles et al., 1993; Yuan and Qian, 2016) and is followed by enzymatic reduction and glycosylation (Mathieu et al., 2005; Leng et al., 2017).

At the end of maturation, and during vinification and wine aging, C13-norisoprenoid compounds are formed in acidic media, through chemical reactions from several volatile and non-volatile precursors (Darriet et al., 2012).

β-ionone (violet-like flavor) and β-damascenone (apple sauce – rose like flavor) are the most ubiquitous C13-norisporenoids, whereas significant quantities of TDN are only found in few cultivars such as Riesling and Ugni blanc, whose wines can have a TDN concentration as high as 200 μg L^−1^ and have a characteristic kerosene/petroleum flavor (Schüttler et al., 2015).

Nine CDD have been identified in Arabidospis, but only *VviCCD1* and *VviCCD4* have been shown to cleave carotenoids at the 9,10 and 9',10' double bonds (Huang et al., 2009; Ahrazem et al., 2010; Lashbrooke et al., 2013). Fifty four genes putatively involved in carotenoid metabolism in *V. vinifera* were identified by Young et al. (2012) and Leng et al. (2017) and seven CCDs have been annotated in grapevine (*VviCCD1.1, VviCCD1.2, VviCCD4a, VviCCD4b, VviCCD4c, VviCCD7*, and *VviCCD8*) (Young et al., 2012; Lashbrooke et al., 2013).

### Fatty Acid Derivates

The unsaturated C_18_ fatty acids linoleic acid and linolenic acid are the precursors of other volatile organic compounds such as C_6_-aldehydes and alcohols like hexanal and hexanol (Kalua and Boss, 2009). Their synthesis occurs mainly before *véraison* in the green berry (Kalua and Boss, 2009) and they have green-grassy aromas even though, considering their detection threshold, they rarely contribute to the herbaceous character of wines. They are formed by the activity of lipoxygenases (*VviLOX*) (Podolyan et al., 2010), *hydroperoxide lyase (VviHPL1 and VviHPL2)* (C_6_-aldehydes and alcohols*-2)* (Zhu et al., 2012), *enal isomerase (3Z)-(2E)*, and *alcohol dehydrogenase (VviADH)* (Kalua and Boss, 2009). The levels of these compounds in wines are mainly modulated by winemaking processes; the more odorous C6 aldehydes (hexanal, hexenals) are reduced to less odorous C6 alcohols by *Saccharomyces cerevisiae* during alcoholic fermentation (Ferreira et al., 1995).

### Methoxypyrazines

**Figure 5:**
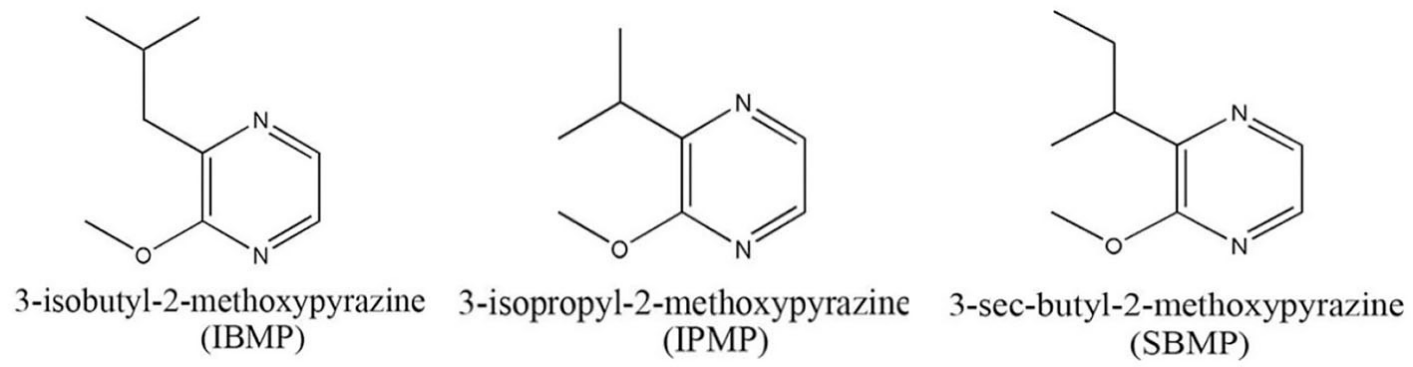
Chemical structures main MPs in grapes.

Methoxypyrazines, nitrogen heterocycle compounds belonging to the pyrazine group, are present in both animals and plants (Maga, 1992). Among the various methoxypyrazines, some alkylated methoxypyrazines, such as 2-methoxy-3-isobutylpyrazine (IBMP), 3-sec-butyl-2-l methoxypyrazine (SBMP), and 2-methoxy-3-isopropylpyrazine (IPMP) (Figure 5) are extremely odorous, with very low odor thresholds (nanogram per liter range in water) (Sidhu et al., 2015). In cultivars of the Carmenet family, such as Sauvignon blanc, Cabernet Sauvignon, Cabernet Franc, Merlot, and Fer, the vegetable-like aromas are reminiscent of pea pods and green peppers, and depending on the concentration they can contribute to earthy nuances (Geffroy et al., 2020). In the grape cluster, the stems contain the largest proportion of IBMP (79.2%), and in the berries, most of the IBMP is located in the skin (72%), followed by seeds (23.8%) and pulp (4.2%) (Roujou-de-Boubée and Botella, 2003).

In grape berries, methoxypyrazines rapidly accumulate between fruit set and the lag phase, peak at 2 to 3 weeks before *véraison*r, then decrease continuously during ripening until harvest (Ryona et al., 2008, 2010; Gregan and Jordan, 2016). The extent to which they are synthesized is still not fully elucidated, whether *in situ* in the berries (Roujou de Boubee et al., 2000; Koch et al., 2010) or in the leaves with subsequent transport via the phloem to the berries where they are degraded (Lei et al., 2018).

Their biosynthesis begins with dicarbonyl addition to the amino acid valine or leucine for IPMP and IBMP and is followed by a methoxylation to form the final methoxypyrazines. In grape, four O-methyltransferases *(VviOMT1-4)* have been characterized, with *VviOMT3* playing a major role in IBMP production (Dunlevy et al., 2013; Guillaumie et al., 2013).

### Thiols

**Figure 6:**
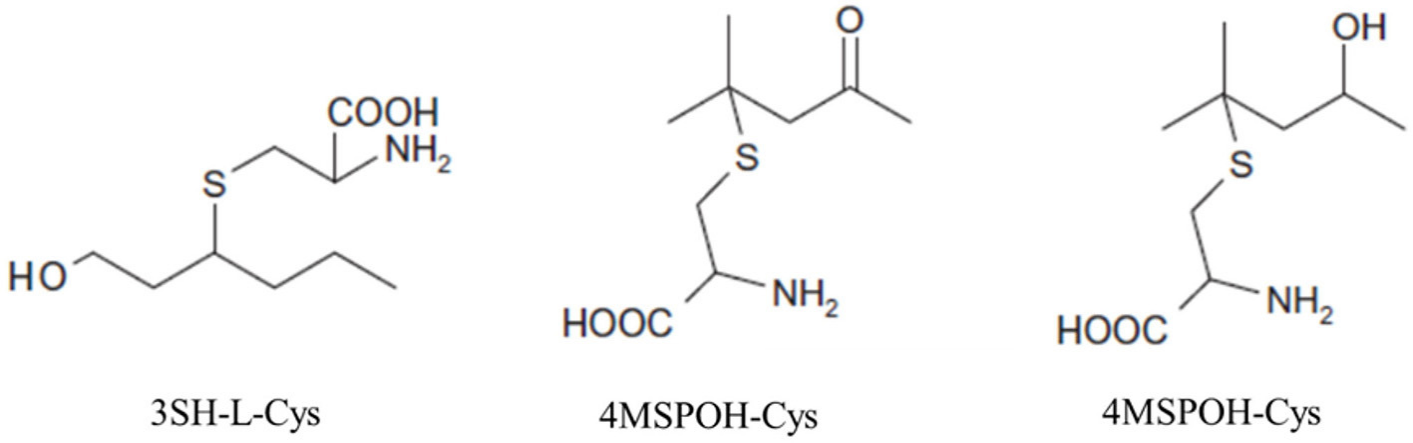
Cysteinylated precursors of 3SH-L-Cys, 4MSPOH-Cys, 4MSP-Cys.

Thiols are volatile sulfur compounds and crucial components of the “varietal character” of several cultivars. 4-methyl-4-sulfanylylpentan-2-one (4MSP), 4-merthyl-4-sulfanylpentan-2-ol (4MSPOH) and the 3- sulfanylhexan-1-ol (3SH) (Figure 6) are the most important thiols present as precursors in the berry of white wine cultivars such as Sauvignon Blanc, Semillon, Petite Arvine, Riesling, Chenin Blanc, Muscat blanc, Colombard, and Alvarino (Tominaga et al., 2000; Fretz et al., 2005), but were found also to contribute to perceived fruitiness in red wines made from Cabernet Sauvignon and Merlot (Bouchilloux et al., 1998). Many of the cultivars known for their high thiol content share parent-offspring or sibling relationships (Duchene et al., 2009b). Thiols are generally linked to flavors reminiscent of passionfruit, box tree, black currant, garlic, and asparagus and are also important volatile components of meat, mushrooms, and many other plants. The first discovered thiol was 4MSP, responsible for aroma of box tree (*Buxus*) (Darriet et al., 1995), followed by the characterization of 3 SH and its ester 3SHA (3-sulfanylhexyl acetate) (Tominaga et al., 1996), often associated with aroma of grapefruit and passionfruit (Tominaga et al., 1998).

Thiols are accumulated during ripening in their non-volatile form, bound to S-cysteine or S-glutathione. The biosynthesis of thiol precursors is linked to glutathione (GSH) metabolism but the biosynthetic pathway of these thiol precursor is not fully elucidated yet. It is related to the conjugation of GHS and α,β-unsaturated carbonyl compounds by S-glutathione transferase as a part of the plant's endogenous metabolism. From this, S-glutathione glutamic acid and glycine is removed resulting in the S-cysteine conjugate. Kobayashi et al. (2011) showed that two genes, *VviGST3* and *VviGST4*, are involved in the production of thiol precursors in grapes.

The localization of different thiol precursors inside the berry is also unclear, and studies often report contradictory results. For example, des Gachons et al. (2005) found that in Sauvignon Blanc grapes at harvest, 4MSP-Cys and 4MSPOH-Cys were localized in the flesh (80%), while 3SH-Cys was equally distributed (50%) between the flesh and the skin. On the contrary, Roland et al. (2011) found that 3SH-Cys and 4MSPOH-Glu were mainly located in the skin (78 and 81%).

The yeast enzyme β-lyase, which is encoded by the gene *IRC7* (Howell et al., 2005; Roncoroni et al., 2011; Santiago and Gardner, 2015), cleaves thiol precursors during alcoholic fermentation. Several other factors, such as active transportation of S-cysteine and S-glutathione conjugates through the plasma membrane, influence thiol release during fermentation and contribute to the varietal expression of thiol-containing cultivars, which can sometimes be considered as too intense and as off-flavors, depending on the concentration.

## Berry Metabolism Mediated by Abiotic Factors

Temperature, water, light, and CO_2_ concentration are among the most important abiotic factors interacting with vine and fruit development in a genotypic-dependent manner (Keller, 2010). These factors are expected to be largely modulated by global climate change. The atmospheric concentration of the greenhouse gas (GHG) CO_2_, the main driver of global warming, has been continuously rising since the beginning of the industrial revolution (around 1,750) due to anthropogenic use of fossil fuel. Concentration increased in the twentieth century from 300 ppm in the early 1960's, when systematic direct atmospheric measurements started (Keeling, 1960), to 400ppm at present time and will, depending on future CO_2_ emission scenarios, reach between 600 (moderate emission scenario) and 1,000 ppm (worst case scenario) by the end of the twenty first century (IPCC, 2013). This entails a rise of temperature, which is expected to be in a range of 2–4.8°C as a function of anthropogenic CO_2_ emissions (IPCC, 2013, 2018). Besides its direct impact on plant physiology, higher temperatures lead to higher evapotranspiration rates and to increased water requirements during the growing season. Together with predicted alterations in precipitation patterns, more severe drought periods can be expected and have already been observed in most viticulture regions (Schultz, 2016). Regarding solar radiation of different wavelengths reaching the earth's surface, predictions of climate change models are ambiguous. Due to its high energy and impact on all living organisms, ultraviolet (UV) radiation (wavelength from 100 to 400 nm) plays a crucial role in the physiology of different plants, mammals and human beings, and terrestrial ecosystems (Ballaré et al., 2011). The UV-B radiation reaching the earth's surface is mostly impacted by the gas composition of the atmosphere. The ozone layer in the upper stratosphere absorbs most of the UV radiation and has been highly degraded by ozone-depleting substances (ODSs) such as chlorofluorocarbons (CFCs) during the last 40 years, which caused a significant increase in UV-B radiation reaching earth's surface. Since the reduction of ODS emissions following the ratification of the Montreal protocol in 1989, the depletion of the ozone layer in the stratosphere was slowed; it remains unclear how UV-B radiation will develop during different climate change scenarios. Certainly the continuous rise of CO_2_ is altering the physical structure of the atmosphere (Chipperfield et al., 2017). Measured concentrations of ozone show an increase of 2–4% per decade, at mid-latitudes and the tropics, in the upper stratosphere (altitude 35–45 km) since about 2000. This increase is consistent with the recovery of stratospheric ozone as a consequence of decreasing concentrations of ODSs and increases in GHGs. In fact, GHGs in the troposphere will isolate upper layers of the atmosphere and a cooler stratosphere which slows down catalytic ozone breakdown (Williamson et al., 2014; United Nations Environment Programme, Environmental Effects Assessment Panel, 2017; Bais et al., 2018, 2019). An additional factor to consider in respect to UV, is its variability due to cloud cover, which often exceeds the variability due to the ozone column depth and is expected to vary in the future due to global climate change as well and remains hard to predict (Enriquez-Alonso et al., 2016).

Altogether the alteration of the different aforementioned abiotic factors will continue to impact viticulture worldwide in different ways. This has triggered discussions among scientists regarding the sustainability of traditional viticulture regions (Fraga et al., 2012, 2013). Different authors predict that the land suitable for viticulture will decrease in main growing regions between 25 and 73% in the future (Hannah et al., 2013), which has however been questioned by other groups as there was no consideration of the capability of cultivars to adapt to changing conditions (van Leeuwen et al., 2013). Recently, more integrated modeling approaches predict that under the most optimistic climate change scenario (2°C increase) and in the most pessimistic scenario (4°C increase) 56 and 85% of current wine growing regions will become climatically unsuitable for viticulture by the end of the twenty first century, respectively (Morales-Castilla et al., 2020). In general, predicted and already observed consequences for wine quality are wines with higher alcohol content, lower acidity, and altered aroma profiles (Schultz, 2000, 2016; Mira de Orduna, 2010; Pons et al., 2017b; van Leeuwen and Destrac-Irvine, 2017) which altogether leads to a loss of typicity and terroir expression (van Leeuwen and Destrac-Irvine, 2017; Van Leeuwen et al., 2018; van Leeuwen et al., 2020).

In the subsequent sections, the most recent and pertinent literature dealing with the impact of the main abiotic factors modified by global climate change on berry metabolism is reviewed.

## Temperature

Temperature is the main driver of vine phenology and its increase advances the vegetative and reproductive cycle of the grapevine and consequently shifts the berry developmental stages toward warmer months of the growing season (van Leeuwen and Destrac-Irvine, 2017). This has been widely confirmed in studies analyzing historical records of harvest dates worldwide which show overall advances of 1–2 weeks over the last several decades (Jones and Davis, 2000; Chuine et al., 2004; Duchêne and Schneider, 2005; Jones et al., 2005; Petrie and Sadras, 2008; Webb et al., 2012; Cook and Wolkovich, 2016). However, crop-yield reductions and evolving management practices may have also contributed to the advancement of ripening (Webb et al., 2013).

The combined effects of advanced phenology and increased temperatures during ripening lead to grapes with higher sugar and less organic acid concentration as well as altered composition in secondary metabolites, such as phenolic and aroma compounds (van Leeuwen and Destrac-Irvine, 2017). In the recent decades, myriad temperature studies have been conducted with a multitude of experimental approaches (Bonada and Sadras, 2015; Gouot et al., 2018). Results are often ambiguous, which makes it difficult to draw general conclusions. Published studies vary from whole plant to bunch level stress applications with variations in the duration of stress application from a couple of hours to several months. A wide range of temperature intensities have been tested from moderate increases (up to 35°C) to severe heat stress (up to 45°C) during day and/or night and at different berry developmental stages. The cultivar and the type of plant material used, as well as the experimental constraints for such studies, affect the responses of berry metabolism to temperature regimes. Control climate chamber experiments are mostly conducted with small model plants such as fruiting cuttings (Pillet et al., 2012; Carbonell-Bejerano et al., 2013; Lecourieux et al., 2017) or mutant microvines (Rienth et al., 2012, 2014a,b, 2016, 2017; Houel et al., 2015; Luchaire et al., 2017; Torregrosa et al., 2017, 2019; Pellegrino et al., 2019).

The dissociation of temperature and radiation effects is difficult to achieve in field trials as compared to climate chamber experiments where heat is applied by convection. However, some early and methodically groundbreaking field experiments succeeded to control clusters' temperature in an radiation-independent way by using different row orientation (Bergqvist et al., 2001), bunch cooling (Spayd et al., 2002) or *in situ* convection systems (Tarara et al., 2008). These studies concluded that some fruit components (in particular anthocyanins) are especially sensitive to temperature (Poni et al., 2018), which to some extent have been subsequently confirmed by molecular studies.

### Temperature Impact on General Fruit Metabolism

Probably the earliest controlled study of the temperature effect on berry metabolism is Ravaz 1912, which inserted single clusters of a vine into a heated glass chamber. Many temperature studies focusing mainly on sugar accumulation, primary metabolisms, and berry growth dynamics have been conducted from the 1960's to the 90's (Kliewer, 1964, 1971, 1977a,b; Kliewer and Lider, 1968, 1970; Buttrose et al., 1971; Kliewer and Torres, 1972; Hale and Buttrose, 1974; Lakso and Kliewer, 1975; Matsui et al., 1986; Sepulveda and Kliewer, 1986) and are summarized in the very first review on the effect of elevated temperature on berry metabolism by Coombe (1987). In summary, moderate temperature increases during ripening hasten berry development and sugar accumulation as observed already in early studies from Buttrose et al. (1971), whichnoted an increase in total soluble solids (TSS) in Cabernet Sauvignon berries when exposed to higher temperatures (30°C). Kliewer and Torres (1972) observed a similar sugar accumulation increase, correlated with increasing temperature until around 35°C, in Cardinal, Pinot noir, and Tokay and Sangiovese even until 41.7°C (Pastore et al., 2017). During early green stages, moderate temperature increases accelerate berry growth, malic and tartaric acid accumulation (Rienth et al., 2016; Arrizabalaga et al., 2018).

When heat stress becomes severe (>35°C), a decrease or arrest of sugar accumulation as well as an inhibition or cessation of berry growth and a delay of ripening is observed (Matsui et al., 1986; Sepulveda and Kliewer, 1986; Greer and Weston, 2010; Greer and Weedon, 2013; Lecourieux et al., 2017; Gouot et al., 2019). This slackening of berry development has been attributed to a lack of carbon supply by photosynthesis, which is impeded and slows down the rate of cell division, which may limit berry size (Keller, 2010). Furthermore, a decrease in ABA biosynthesis as indicated by the repression of *VviNCED2* and *VviNCED4* transcripts and the upregulation of *VviABI3* a B3-domain TF that is a part of the core ABA signaling network were associated with slower berry growth (Rienth et al., 2014b; Lecourieux et al., 2017). In addition, the role of auxins biosynthesis and regulation, which inhibits berry growth and sugar and anthocyanin accumulation (Davies et al., 1997; Bottcher et al., 2010) could putatively contribute to the delay of berry development and onset of ripening under severe heat stress as evidenced by the reduction of IAA-amido synthetase (*VviGH3*) and an increase in the expression of transcripts encoding IAA-amino acid hydrolases (Lecourieux et al., 2017).

The slackening of sugar accumulation rates under heat stress has been correlated with a down-regulation of sugar transporters (STPs) and invertase-encoding genes (*GIN2*) putatively involved in the import and accumulation of hexoses into vacuoles. The latter genes were found to be repressed in fruiting cuttings of Cabernet Sauvignon (Lecourieux et al., 2017), Muscat Hamburg (Carbonell-Bejerano et al., 2013) and microvines (Rienth et al., 2014b) upon exposure to high temperature. Curiously, a recent field study using an open top heating system found an altered glucose / fructose ratio due to lower glucose concentration in Riesling berries under high temperature (+10°C) (Brandt et al., 2019). Similar observations have been reported in historical studies and attributed the altered glucose/fructose ratio to over-ripeness of grapes (Kliewer, 1965, 1967; Sepulveda and Kliewer, 1986). This has, however, not been observed or closely investigated in other heat stress studies (Carbonell-Bejerano et al., 2013; Sweetman et al., 2014; Lecourieux et al., 2017).

Drawing upon the results described above, it seems that the most sensitive berry stage to biotic and abiotic stress is just around or during the *véraison* phase as evidenced by a couple of studies with precise single berry sampling protocols (Rienth et al., 2014b, 2016; Torregrosa et al., 2019; Ghaffari et al., 2020).

Concerning malic and tartaric acid, the two main organic acids in grape berries, it appears that moderately increased temperature during early berry development from anthesis to lag phase accelerates their accumulation (Sweetman et al., 2014; Rienth et al., 2016; Lecourieux et al., 2017). However, when heat stress becomes too severe, their synthesis is inhibited, as reported, for example, by Gouot et al. (2019), where temperatures reached up to 45°C during early green growth.

It has been shown in myriad historic and recent studies that high temperatures are correlated with a decrease in malic acid concentration in the ripening berry (Ruffner et al., 1976; Ruffner and Hawker, 1977; Possner et al., 1983; Sweetman et al., 2009, 2014; Carbonell-Bejerano et al., 2013; Etienne et al., 2013; Rienth et al., 2016; Lecourieux et al., 2017; Brandt et al., 2019). This degradation is highly cultivar-dependent and can even vary among clones, as shown for example on different Tempranillo clones exposed to high temperatures (Torres et al., 2017). However, the underlying regulation of malic acid degradation remains to be elucidated. Sweetman et al. (2014) found that high temperatures accelerated the NAD dependent malic enzyme activity and decreased phosphoenolpyruvate carboxylase and pyruvate kinase activities, accompanied by the accumulation of various amino acids and γ-aminobutyric acid, suggesting an enhanced anaplerotic capacity of the TCA cycle and a need to deal with decreased cytosolic pH in heated berries. This study also found differences depending on the diurnal temperature range: that is, the loss of malic acid content in response to elevated day temperature was lessened if night temperature was also increased. It was also proposed that malate concentration is mainly depends on the thermodynamics of its tonoplastic transport (Lobit et al., 2006).

It is generally reported that tartaric acid (TA) is not metabolized by the ripening berry and thus its content is not impacted by temperature and represents an important trait for breeding programs seeking new cultivars better adapted to future climate conditions (Duchêne, 2016). Observed variation of TA in the scientific literature (Cholet et al., 2016) is a likely consequence of dilution effects or precipitation during sample processing (Rösti et al., 2018).

### Temperature Impact on Phenolic Compounds

The direct and indirect effects of temperature on flavonoid composition are far from being completely unambiguous. In this section we summarize and update recent findings.

Temperature effects on flavonol and flavan-3-ol composition are not always consistent among studies (Gouot et al., 2018). However, there is unambiguous scientific evidence that shows deleterious effects of high temperature on anthocyanin levels in the grape berry. This was reported in early studies (Buttrose et al., 1971; Kliewer and Torres, 1972; Spayd et al., 2002) and more recently confirmed by physiological and molecular studies (Mori et al., 2005, 2007; Yamane et al., 2006; Azuma et al., 2012; Carbonell-Bejerano et al., 2013; Rienth et al., 2014b; Lecourieux et al., 2017; Pastore et al., 2017; Torres et al., 2017; Yan et al., 2020). Heat stress has been shown to repress major anthocyanin biosynthesis regulators such as *VviMYBA1* and downstream genes such as *VviUFGT, VviCHI, VviF3H2, VviDFR*, and *VviLDOX*. However, not all of these studies showed unequivocal repression nor a correlation with lower anthocyanin accumulation. This is possibly due to the use of different plant materials (normal vines, different cultivars), different stages of berry development and intensities of treatment, and sampling strategy. Furthermore, the temperature effect on anthocyanin synthesis varies highly between genotypes. For example, when the temperature maximum during ripening exceeded 35°C, inhibition of color formation was much more pronounced in Grenache than in Carignan (Fernandes de Oliveira et al., 2015). In earlier studies, low temperature during ripening, in particular at night was associated with enhanced coloration of grapes (Kliewer and Torres, 1972), which was confirmed in a recent molecular experiment where low night temperatures enhanced anthocyanin accumulation and expression of *VviCHS3, VviF3H1, VviUFGT*, and *VviMYBA1*, in particular when applied around *véraison* on Corvina grapes (Gaiotti et al., 2018). In Kyoho grapes, temperature increases from 27 to 30°C during ripening induced a strong decrease of the transcript levels of anthocyanin genes, leading to less berry color (Shinomiya et al., 2015). In Merlot, an increase of day temperature of 5°C during ripening, from 20 to 25°C, resulted in a anthocyanin decrease of 37% (Yan et al., 2020). Besides the repression of anthocyanin related genes, high temperature may promote anthocyanin degradation, possibly via the increased activity of peroxidases (Mori et al., 2007). This is evidenced by the upregulation of a gene coding for a peroxidase, *VviPrx31*, in berries exposed to high temperature (Movahed et al., 2016), and a similar effect occurs in other plant species, such as *Brunfelsia* flower petals (Vaknin et al., 2005), litchi (Zhang et al., 2005), and strawberry fruits (Chisari et al., 2007).

An increased proportion of acylated and tri-hydroxylated anthocyanins under higher temperature has been observed in experiments with Merlot (Tarara et al., 2008; Yan et al., 2020), Cabernet Sauvignon (Lecourieux et al., 2017), Sangiovese fruiting cuttings (Pastore et al., 2017), and Malbec (de Rosas et al., 2017), concomitantly with the overexpression of the acyltransferase gene *Vvi3AT* (de Rosas et al., 2017; Yan et al., 2020). Similarly for anthocyanins, high temperature impeded flavanol accumulation strongly and increased methoxylated (isorhamnetin and syringetin) and 3′, 4′, 5′-substituted (myricetin and syringetin) flavonols in Merlot (Yan et al., 2020).

Interestingly, high temperature can cause an uncoupling of sugar accumulation and anthocyanin synthesis leading to a lower anthocyanin / sugar ratio, possibly because of a delay in the onset of anthocyanin synthesis (Sadras and Moran, 2012; Sadras et al., 2013; Yan et al., 2020) or a reduced anthocyanin accumulation during ripening (Yan et al., 2020). The magnitude of this thermal decoupling is cultivar dependent as shown for Grenache and Carignan (Fernandes de Oliveira et al., 2015) and can vary between clones of the same cultivar as shown in Tempranillo (Arrizabalaga et al., 2018).

The effect of temperature on tannins is still not well-understood. The synthesis of the tannin monomers, flavan-3-ols, was increased under elevated temperature (Cohen et al., 2012a,b), although in both latter studies, differences were no longer significant at *véraison*. Tannins were not affected by heat stress in Sangiovese (Pastore et al., 2017), as well as in the study of Gouot et al. (2019) who reported an absence of effect on flavan-3-ol or tannin levels, but a significantly higher percent of galloylation of flavan-3-ols, consistently with that reported by Cohen et al. (2012a) and indicated bythe overexpression of *UDP glucose-gallic acid-glucosyltransferase* under high temperature as reported by Rienth et al. (2016).

With regards to stilbene synthesis, heat stress inhibited the expression of members of the STS biosynthetic pathway (Rienth et al., 2014b), while grape exposure to low temperature upregulated STS transcripts indicating a higher stilbene biosynthesis at low temperature (Pastore et al., 2017).

### Temperature Impact on Aroma Compounds

In plants, high temperature causes an increase in aroma compound production and emission as shown particularly for terpenes up to a certain threshold, generally around 40°C (Guenther et al., 1993; Copolovici and Niinemets, 2016). In grape berries, the temperature impact on aroma compound accumulation is ambiguous (Selmar and Kleinwächter, 2013; Lecourieux et al., 2017). From recent studies, it appears that the aroma levels are reduced when heat stress is applied both at the cluster scale (Lecourieux et al., 2017) and at the whole plant scale if stress is applied during berry ripening (Rienth et al., 2014b). In Sangiovese, Pastore et al. (2017) reported an increase in TPS expression under higher temperature before ripening and a repression of linalool synthase, delta-cadinene synthase, vetispiradiene synthase, and a germacrene enzyme activity during ripening. In Sauvignon blanc exposed to higher berry temperature through leaf removal, the concentration of thiol precursors in berries at harvest was not significantly modified (Sivilotti et al., 2017). However, Wu et al. (2019) found a lower concentration of the aldehydic glutathionylated precursor of 3SH (Glut-3SH-Al) in grapes from Cabernet Sauvignon and Sauvignon Blanc under a 1.5°C temperature increase.

Temperature studies on carotenoids, the precursors of C13 norisoprenoids (see above), are also inconsistent, most likely because of the difficulty in separating temperature and radiation effects in field trials. Higher carotenoid levels were found in Riesling and Chenin Blanc grown in warmer than cooler regions (Marais et al., 1991). In the Duoro Valley in Portugal, cooler temperature at higher altitudes possibly contributed to the lower berry carotenoids concentration in Portuguese autochthonous cultivars (Oliveira et al., 2004). Fernandes de Oliveira et al. (2015) found consistently higher carotenoid content in shaded grapes compared to grapes exposed to direct sunlight. However, leaf removal in the fruiting zone, which generally increases temperature around berries, did not alter total carotenoids in Riesling (Kwasniewski et al., 2010).

The concentration of the C13 norisoprenoid 1,1,6-trimethyl-1,2-dihydronaphtalene (TDN), which determines the kerosene-like notes of aged Riesling, is generally higher in warm climates (Marais et al., 1992; Schüttler et al., 2015). Carotenoid and C13 norisoprenoid related transcripts, such as phytoene synthase (*VviPSY*), which catalyzes the first step of carotenoid biosynthesis, and downstream genes coding the phytoene dehydrogenase, carotenes desaturase, carotenoid isomerase, lycopene cyclase, and carotene hydroxylase were concomitantly downregulated by vine exposure to high temperature (Rienth et al., 2014b; Lecourieux et al., 2017).

Considering the cultivars that produce MPs, wines produced in cooler regions have higher MP concentrations and display a more vegetative odor, as shown for Cabernet Sauvignon and Sauvignon Blanc (Allen et al., 1991; Falcão et al., 2007). However, it is still not clear to what extent temperature contributes to the decrease of MPs before and during ripening, and to what extent the decrease is due to the radiation (Darriet et al., 2012; Lei et al., 2018). The limited molecular data indicates a repression of IBMP synthesis already in the green berry exposed to high temperature as highlighted by the repression of the key gene *VviOMT3* during heat stress experiments with fruiting cuttings (Lecourieux et al., 2017).

Recent research has characterized other aroma components contributing to overripe aromatic nuances in grapes and wines of red cultivars (i.e., nuances of jammy fruit, and prune in Merlot and Cabernet Sauvignon wines) (Pons et al., 2017a; Allamy et al., 2018). Such grapes and wines presented a greater concentration of chemical compounds belonging to the ketones, furanones and lactones family. Moreover, the analysis of wines of different vintages from a Pomerol estate contained higher concentrations of 5,6-dihydro-6-pentyl-2-(2H)-pyranone (called massoia lactone or 2-decen-5-olide) and gamma-non-alactone during vintages with higher average temperatures. This leads to the hypothesis that increased temperatures due to climate change will increase the perception of overripe fruity notes, as occurring now in hot vintages (Pons et al., 2017a).

## Radiation

As described above, changes in the gas composition of the atmosphere are forecasted for the future. These changes may affect the amount and composition of solar radiation reaching the earth's surface. The exposure of grapes to sunlight is normally associated with higher berry quality due to higher levels of total soluble solids, anthocyanins, and phenolics in general, and lower levels of acids and juice pH as well as lower incidence of disease due to better microclimates (Dokoozlian and Kliewer, 1996; Bergqvist et al., 2001; Abeysinghe et al., 2019).

### Impact of Radiation on Phenolic Compounds

The exposure of grapes to sunlight normally increases the level of phenolic compounds as shown, for example, in Riesling (Brandt et al., 2019), Pinot Noir (Song et al., 2015), Summer Black (Xi et al., 2016), and Cabernet Sauvignon (Blancquaert et al., 2019). In parallel, sunlight exposure enhances the expression of structural and regulatory phenylpropanoid genes (Chorti et al., 2010; Matsuyama et al., 2014; Wu et al., 2014; Friedel et al., 2015; Sun et al., 2017). Among phenolics, the most light-responsive ones are flavonoids and in particular flavonol glucosides, whose levels increase dramatically with increasing sunlight exposure, consistently with their UV radiation-screening activity and their capacity to reduce light-induced oxidative damage (Downey et al., 2004; Matus et al., 2009; Agati et al., 2013; Martínez-Lüscher et al., 2014; Reshef et al., 2017, 2018). In a recent comprehensive study that used leaf thinning and shoot removal in Cabernet Sauvignon and Petit Verdot to improve sun exposure, the flavonols kaempferol, quercetin, and myricetin significantly increased, whereas no changes of other flavonoid compounds occurred (Torres et al., 2020a). Alike, Sun et al. (2017) found higher hydroxycinnamic acids and flavonol levels in Cabernet Sauvignon when sun exposure was increasd.

Several transcriptomic studies indicate that, in the berry skin, flavonol genes – for example *VviFLS1, VviGT5* and *VviGT6* in Tempranillo berries – are more induced than those of other phenylpropanoids upon UV radiation (Koyama et al., 2012; Loyola et al., 2016). Reciprocally, Matus et al. (2009) found reduced expression of *VviFLS4* and its putative transcriptional regulator *MYB12* when berries were shaded.

It is not fully understood to what extent visible and/or UV light contribute to the stimulation of the synthesis of phenolic compounds (Keller and Torres-Martinez, 2004; Schreiner et al., 2012; Teixeira et al., 2013). Drawing upon recent studies, it seems that particularly the UV-B fraction of solar radiation is responsible for the enhanced expression of key flavonoid genes (Koyama et al., 2012; Teixeira et al., 2013; Carbonell-Bejerano et al., 2014; Martínez-Lüscher et al., 2014; Liu et al., 2015; Loyola et al., 2016). Recently, two bZIP TFs elongated hypocotyl 5 protein (HY5) orthologs, *VviHY5* and *VviHYH*, were characterized as constituents of the UV-B response pathway in grapevine and mediated flavonol accumulation in response to high radiation exposure (Loyola et al., 2016; Matus et al., 2017).

The exposure of grape clusters to light significantly increases anthocyanin accumulation, whereas shading reduces it (Spayd et al., 2002; Downey et al., 2004; Matus et al., 2009; Song et al., 2015; Guan et al., 2016). In a comprehensive *in vitro* study that considered the effect of berry exposure to light and temperature treatments, Azuma et al. (2012) reported higher anthocyanin levels in grapes exposed to higher light levels. These higher levels correlated with the upregulation of most genes of the anthocyanin biosynthesis pathways. Several other studies confirm the induction of major anthocyanin genes such as the TF *VviMYBAa* together with *VviUFGT* under elevated sun exposure (Koyama et al., 2012; Shinomiya et al., 2015). Interestingly, UV-B radiation induces the expression of *VviMYBA1* and significantly delays the down-regulation of *VviMYBA6* and *VviMYBA7* at the latter stages of berry development (Matus et al., 2017).

Low light conditions modulate the proportion of di- to tri-hydroxylated anthocyanins more toward tri-hydroxylated anthocyanins as evidenced by the downregulation of *VviF3*′*5*′*Hs* (Azuma et al., 2012; Koyama et al., 2012; Guan et al., 2016). Similar trends, but inconstent amongst years, have been recently reported in warm climates with Cabernet Sauvignon (Sun et al., 2017) and Petite Verdot (Torres et al., 2020a,b). It seems that low light conditions increase the concentration of non-acylated anthocyanins (Downey et al., 2006; Matus et al., 2009) but this remains to be confirmed in future studies.

Recently, a role of miRNA on the anthocyanin response to UV-B radiation has been suggested. Sunitha et al. (2019) hypothesize that the UV-B induced upregulation of miR3627/4376 facilitates anthocyanin accumulation by antagonizing a calcium effector, and showed that miR395 and miR399, which are induced by micronutrient deficiencies also known to trigger anthocyanin accumulation, respond positively to UV-B radiation. Finally, increases in the abundance of the MYB-bHLH-WD40 complex, which regulates anthocyanin production, are mediated by UV-B-induced changes in miR156/miR535. The same changes could contribute to the observed up-regulation of miR828. In turn, miR828 would regulate the AtMYB113-ortologues *MYBA5, A6* and *A7* (and thereby anthocyanins) via a widely conserved and previously validated auto-regulatory loop involving miR828 and phasi TAS4abc RNAs.

### Impact of Radiation on Aroma Compounds

It is generally known that light exposure increases the concentration of most aroma compounds in grape berries, but that excessive sunlight as well as its total exclusion inhibits the accumulation of most aroma compounds (Bureau et al., 2000; Zhang et al., 2014; Young et al., 2016).

Because carotenoids are pigments with photoprotective function, their biosynthesis is generally enhanced under high radiation as are their degradation products. The levels of C13 norisoprenoids are as well highly correlated with extended sun exposure (Kwasniewski et al., 2010; Schüttler et al., 2015; Young et al., 2016). Sunlight exposure increased the concentration of β-ionone, but the increase was not statistically significant for UV treatment (Song et al., 2015), and TDN is typically found in high amounts in sun-exposed Riesling berries, potentially resulting in a higher petrol aroma of wines (Mendes-Pinto, 2009).

Young et al. (2016) reported that the cytosolic CCD1-encoding genes were up-regulated in exposed clusters in the earlier stages of berry development. Conversely, the chloroplastic CCD4-encoding genes were down-regulated in exposed clusters during ripening. Therefore, Young et al. (2016) suggested that the increased levels of norisoprenoids observed in exposed berries during ripening were not due to increased gene expression (of the CCD4-encoding genes) but rather due to increased substrate (carotenoid) availability.

Monoterpenes, and particularly linalool, are highly sensitive to sunlight, as shown, for example, in Sauvignon Blanc and Riesling (Sasaki et al., 2016), where the expression of *VviDXS* and of linalool synthases was reduced by low sun exposure and by UV-B exclusion, resulting in lower levels of linalool content. Similarly, Zhang et al. (2017) showed that, in the Muscat variety Jingxiangyu, linalool was the most sensitive compound to sunlight followed by ocimene and glycosylated geraniol. The reduction in the levels of these terpenes by sunlight exclusion correlated with the reduced expression of *VviPNLinNer1, VviCSbOci, VviGT7*, and *VviGT14* genes. Similarly, Carbonell-Bejerano et al. (2014) reported an upregulation of genes involved in monoterpenoid biosynthesis, such as 1,8-cineole/eucalyptol synthase and two linalool synthases, in Tempranillo berries exposed to high UV-B radiation. In general, monoterpene levels were induced by exposing clusters to sunlight (Song et al., 2015; Feng et al., 2017).

Young et al. (2016) showed that increased photosynthetically active radiation (PAR) (+52%) led to higher levels of volatile terpenoids in the exposed Sauvignon Blanc berries; however, there were clear differences in the responses based on the developmental stage considered.

Similarly, Šuklje et al. (2014) found increased concentration of thiols in clusters exposed to sunlight, and it is generally known that light exposure during ripening reduces MP content in berries (Roujou de Boubee et al., 2000; Sala et al., 2004; Stummer et al., 2005; Darriet et al., 2012; Sidhu et al., 2015; Martin et al., 2016; Cassandra et al., 2019; Torres et al., 2020a). Interestingly, according to Koch et al. (2012), higher light intensity before and not during ripening has a greater impact of methoxypyrazine concentration at harvest. Dunlevy et al. (2013) showed that both the precursor of 3-isobutyl-2-hydroxypyrazine (IBHP) and the expression of *VviOMT3*, a gene that controls the final step of methoxypyrazine biosynthesis, were drastically reduced in exposed clusters(Koch et al., 2012).

Allamy et al. (2018) considered separately the specific impact of light exposure on berry aroma compounds during post-harvest storage. Under conditions of light exposure, significant increases in furaneol, homofuraneol and γ-nonalactone concentrations were noticed in both grape juice and wine (Allamy et al., 2018).

## Water

### Impact of Vine Water Status on Phenolic Compounds

Studies investigating the effect of water availability on berry physiology and quality have been recently reviewed by Scholasch and Rienth (2019), Rienth and Scholasch (2019), and Gambetta et al. (2020). Impact of water deficit on berry development depends on the intensity and duration of the stress as well as the developmental stage. Water deficit stress during the first growth phase had the highest impact on final berry volume and consequently yield. Water deficit reduces cell expansion without impacting the rate of cell division (Ojeda et al., 2001), contrary to frequent speculations. During the ripening phase, water deficit has a smaller impact on berry size than during the first growth phase, probably because of impaired hydraulic connections with the parental plant.

It is generally known that a moderate water deficit (predawn leaf water potential between−0.3 to −0.5 MPa) is beneficial for final wine quality which is in particularly true for red cultivars (Van Leeuwen et al., 2009; Zufferey et al., 2017). Because water deficit reduced berry volume, positive effects can partly be attributed to higher concentration of quality determining compounds synthesized in the skin cells (the surface of the berry). However, an enhanced accumulation of secondary metabolites independently of berry volume changes has been already highlighted by Ojeda et al. (2002) and confirmed by several molecular studies that observe an upregulation of key enzymes of the phenylpropanoid and flavonoid pathways (Castellarin et al., 2007a,b; Cramer et al., 2007; Deluc et al., 2009, 2011; Teixeira et al., 2013; Savoi et al., 2016, 2017; Zarrouk et al., 2016a,b).

The most beneficial effects are observed when water deficit occurs throughout ripening. Aside from an overall increase in the accumulation of phenylpropanoids and flavonoids (Chorti et al., 2016; Koundouras, 2018), several studies showed a modification in composition of anthocyanins toward a relative increase of tri-hydroxylated anthocyanins (3′,4′,5′-hydroxylated: delphinidin, petunidin, malvidin) (Castellarin et al., 2007a; Ollé et al., 2011; Cook et al., 2015). However, the changes in the anthocyanin profile in response to water deficit appear to be highly varietal dependent (Niculcea et al., 2014; Theodorou et al., 2019). Some reports showed an increase proanthocyanidin concentration and proanthocyanidin polymerization levels in grape berry skins (Kyraleou et al., 2016; Cáceres-Mella et al., 2017), and higher catechin levels (Zsófi et al., 2014). The observed increase of phenolic compounds when water deficit occurs prior to *véraison* can mainly be attributed to concentration effects (Santesteban et al., 2011; Brillante et al., 2018); however, several studies also observed an increase in anthocyanin content per berry (Ojeda et al., 2002; Castellarin et al., 2007a; Koundouras et al., 2009; Ollé et al., 2011).

### Impact of Vine Water Status on Aroma Compounds

Reported effects of water availability on aroma compounds are less evident than for phenolic compounds. Most water deficit studies on grape aroma compounds show very heterogeneous results, depending on the type of aroma compounds considered, as reviewed by Alem et al. (2019). A positive relationship between increasing water deficit and the concentration of C13-norisoprenoids such as β-damascenone, β-ionone, and 1,1,6-trimethyl-1,2-dihydronaphthalene was reported. This was particularly true for red cultivars such as Cabernet Sauvignon (Bindon et al., 2007; Koundouras et al., 2009; Brillante et al., 2018), Merlot (Song et al., 2012), and Tempranillo (Talaverano et al., 2017). In of Talaverano et al. (2017), C6 compounds (hexanal, trans-2-hexenal, and 1-hexanol), phenol volatiles, ethyl esters, and lactones were also found to be increased under water deficit, as opposed to Song et al. (2012), which reported a decrease of those compounds under water deficit in Merlot.

Several studies reported increased concentrations of monoterpenes such as limonene, linalool, α -terpineol geranyl acetone, geraniol, and citronellol under light to moderate water stress (Savoi et al., 2016; Brillante et al., 2018; Wang et al., 2019; Kovalenko et al., 2021), which was associated with increased expressions of terpenoid synthases-genes in Chardonnay and Tocai Friulano (Deluc et al., 2011; Savoi et al., 2016). Some authors report higher monoterpene concentrations, even under severe water deficit (Schüttler et al., 2015). One of the few aroma compounds whose concentration in the berry is negatively correlated with moderate water deficit is the recently discovered sesquiterpene rotundone (Wood et al., 2008; Geffroy et al., 2014, 2018) associated with notes of black pepper.

The precursors of volatile thiols (S, 4MSPOH and the 3SH) present in the berry (Tominaga et al., 2000; Fretz et al., 2005) increased under mild water deficit and decreased under severe stress (predawn leaf water potential close to −1.0 MPa) (des Gachons et al., 2005). However, since nitrogen is very important for the production of volatile thiols of grapes (Helwi et al., 2015, 2016) and its absorption can be limited by water deficit (Celette and Gary, 2013), the effects observed may be indirect and largely due to a limited nitrogen absorption. Picard et al. (2017) found that the exposure of vines to water deficit positively relates to the perception of aging bouquet typicality (truffle and underwood aroma) in premium Bordeaux wines. It appears that moderate water deficit increases the concentration of C13-norisoprenoids most likely from higher sun exposure due to reduced canopy (Koundouras et al., 2009). On the other hand, the reduced canopy caused by water deficit might favor methoxypyrazine degradation (Brillante et al. (2018) and Harris et al. (2012).

## Co_2_ Concentration

Elevated carbon dioxide (eCO_2_) concentration is generally beneficial for plants because it leads to increases in the rate of photosynthetic carbon fixation by leaves. This leads primarily to increased plant growth and biomass production and translates into increases in harvestable yield of wheat, rice and soybean, all showing 12–14% yield increases under eCO_2_ in FACE (Free Air Carbon enrichment) experiments (Ainsworth and Long, 2005; Ainsworth and Rogers, 2007; Ainsworth, 2008). In fruit crops and vegetables, eCO_2_ generally increased the total antioxidant capacity as well as the concentrations of fructose, glucose, total soluble sugars, total phenolics, total flavonoids, ascorbic acid, and calcium in the edible parts (Sun et al., 2017).

Most studies on grapevine dealing with eCO_2_ focused on vegetative growth and photosynthesis while physiological and molecular studies on berry metabolism are relatively scarce so far. All studies report increased photosynthesis leading to a yield and biomass increase under eCO_2_ (Goncalves et al., 2009; Moutinho-Pereirea et al., 2009; Kizildeniz et al., 2015; Edwards et al., 2016, 2017; Wohlfahrt et al., 2018). In climate chamber studies, Martinez-Luscher et al. (2015) highlighted the dependence of berry ripening rates on the carbon fixation process which is correlated to CO_2_ concentration.

Only some grape attributes have been found to be affected by eCO_2_. In particular, sugars, acids, and berry size increased under eCO_2_ (Bindi et al., 2001; Kizildeniz et al., 2015). However, in a FACE study that considered Riesling and Cabernet Sauvignon vines exposed to moderate increases of atmospheric CO_2_, sugar concentration was not affected even though yields were increased (Wohlfahrt et al., 2018). In wines made from the latter FACE experiment must and young wines quality and compositin was not found to be negatively influenced by an eCO_2_ (Wohlfahrt et al., 2021). Anthocyanins and proanthocyanidins were not affected by eCO_2_ in most studies (Goncalves et al., 2009; Salazar-Parra et al., 2012; Kizildeniz et al., 2015).

In multistress experiments with Temperanillo fruiting cuttings where future temperature (+4°C) and CO_2_ (700 ppm) conditions where simulated, high CO_2_ in particular when combined with high temperature hastened berry ripening, sugar accumulation, malic acid respiration and reduced the aforementioned high temperature induced anthocyanin–sugar decoupling (Arrizabalaga-Arriazu et al., 2020a).

The effect of eCO_2_ on aroma compounds remains to be elucidated. Goncalves et al. (2009) found no impact on aroma compounds such as C6 alcohols, alcohols, esters, terpenols, carbonyl compounds, acids, volatile phenols, and C13 norisoprenoids under moderate eCO_2_ (500 ppm). However, the same treatment induced an increased level of ethyl 2-methylbutyrate, isoamyl acetate, ethyl hexanoate, and ethyl octanoate.

## Biases Generated By Berry Heterogeneity and Volume Variation

Empirically, winegrowers and berry physiologists know that grape ripening is a very heterogenous process which can easily by visually perceived around *véraison* when, in red grape cultivars, berries of the same cluster change color asynchronously. For “fine scale” physiological studies that aim to reveal the effect of climate and abiotic factors on grape berry composition, the random time scale of berry sampling that is commonly adopted can cause important biases in chemical composition and gene expression data, which can potentially mask important physiological information. Poor correlation of phenotypic and transcriptomic data often observed in abiotic and biotic stress studies can be partially attributed to berry heterogeneity and sampling strategy.

Several molecular studies tried to address the complex issue of grape ripening on a single berry scale (Gouthu et al., 2014; Rienth et al., 2014b, 2016; Shahood et al., 2015, 2020; Carbonell-Bejerano et al., 2016; Rösti et al., 2018). For instance, Carbonell-Bejerano et al. (2016) performed berry density sorting by berry flotation in NaCl solutions and assessed the transcriptome of different berry ripening groups and showed that gene expression profiles clearly relate with ripening progression of different sorted berry groups. By contrast, when the same density series were sampled on two different dates from the same vineyard of Tempranillo, considerable differences were detected, which indicated that environmental differences between both sampling moments determined most of these expression differences. Latter findings highlight evidence on the convenience of a homogenization of the developmental stage and the sampling time condition for transcriptome comparisons by berry density sorting.

Previous heat stress studies using RNA from single berries sorted according to their biochemical characteristics led to the discovery of anthocyanin biosynthesis related transcripts (Rienth et al., 2014b) that have not been detected in other studies. In several studies (Carbonell-Bejerano et al., 2013; Lecourieux et al., 2017; Pastore et al., 2017), anthocyanin concentration was significantly decreased by heat stress, but the expression pattern of key anthocyanin regulator genes such as *VviMybA1* and *VviAOMT* did not show concomitant repressions as opposed to the heat stress study of Rienth et al. (2014a,b), where berry batches where constructed according to berry biochemical characteristics. Similar discrepancies in gene expression data were observed in molecular studies conducted on berries of virus infected vines and emphasize the heterogeneity problem, where the gene expression results changed if berries were sampled at same calendar day without sorting (Vega et al., 2011) or if individual berries were grouped according to their “internal ripening clock” (Ghaffari et al., 2020). The “highest precision” is in our opinion obtained by single berry analysis of sugar and acids and a subsequent grouping of berries according to those to parameters. Obviously, such sampling strategies are labor intensive and above all time consuming. A good compromise could be berry sorting by density, which, however, will require some time for the sorting before the berries can be frozen in liquid nitrogen, during which time the transcriptome of the abscised berry may evolve leading to biases. An alternative, less time-consuming, sampling approach is to measure sugar concentration of each berry prior to N-freezing as adopted in a recent study by Cramer et al. (2020). Researchers need to weigh the advantages and drawback of different sampling strategies for each experiment and when interpreting their results.

Another potential issue is related to how the levels of compounds are expressed. This can prevent researchers from drawing clear physiological conclusions. Commonly, metabolite (sugars, acids, phenolic compounds, etc.) levels in the grape berry are expressed as concentration, i.e., amount per volume (L) or weight (g), because the metabolite concentration in the wine is more closely related to the metabolite concentration in the berry than to the total metabolite amount in the berry. The metabolite concentration depends on the amount accumulated in the cells and the berry volume which change dramatically during development and/or due to responses to abiotic stresses (e.g., limited water availability that decreases berry size). Therefore, expressing metabolites as concentrations can lead to misinterpretations of the effects of specific treatments or stresses on the metabolic responses of the berry (Famiani et al., 2015; Moscatello et al., 2019). Thus, expressing both the concentration and the amount per berry of a given metabolite allows for a better identification of direct and indirect effects of treatments/factors on such metabolite.

## Conclusion

A changing climate requires a profound knowledge of how abiotic factors modulate different quality-determining compounds of the grape berry in order to implement appropriate viticultural mitigation strategies (van Leeuwen and Destrac-Irvine, 2017; Rienth et al., 2020), select varieties, clones (Wolkovich et al., 2018), and rootstocks (Ollat et al., 2016), and to identify traits, genes, or QTLs for the breeding of new cultivars better adapted to future conditions (Duchêne, 2016).

The physiological and molecular knowledge of the mechanisms involved in the biosynthesis and degradation of secondary metabolites in the grape berry has significantly increased in the past two decades and is continuously advancing due to the development and improvement of omic tools. However, the impact of environmental factors, notably light and temperature, is often ambiguous (van Leeuwen et al., 2020). This can partly be attributed to the difficulty in separating light and temperature effects and the interaction between the two. A further often neglected bottleneck in physiological studies on grape berry responses to climate and abiotic factors is the effect of heterogeneity and the aforementioned berry sampling strategy, which may impair our capability to analyses complex metabolic events. Post-transcriptional and epigenetic regulation of metabolism, which have so far rarely been addressed in physiological studies, might play important roles in the endogenous and exogenous modulation of secondary metabolism in the grape berry.

Facing global warming, viticultural practices such as cluster and shoot thinning or leaf removal, historically considered as improving quality, need to be reconsidered and adapted to changing conditions (van Leeuwen and Destrac-Irvine, 2017; Torres et al., 2020a,b). Moreover, field data on the impact of increased atmospheric CO_2_ concentration on berry metabolism are scarce and need to be further investigated, in particular in combination with different abiotic stresses such as increased temperature and drought. Though difficult to conduct in field conditions and so far only carried out on fruiting cuttings (Salazar-Parra et al., 2012; Kizildeniz et al., 2015; Martinez-Luscher et al., 2015; Arrizabalaga-Arriazu et al., 2020a,b) such multi-stress experiments will improve understanding of how climate change will impact vine and berry physiology, and will help develop mitigation strategies.

## Data Availability Statement

The original contributions presented in the study are included in the article/supplementary material, further inquiries can be directed to the corresponding author/s.

## Author Contributions

MR and SDC devised the main body and structure and content of the manuscript. PD, CS, CBu, CBo, NV, RW, and FF provided valuable ideas and triggered fruitful discussions via helpful comments and provided corrections. All others approved the final version of the manuscript.

## Conflict of Interest

The authors declare that the research was conducted in the absence of any commercial or financial relationships that could be construed as a potential conflict of interest. The reviewer ZD declared a past co-authorship with one of the authors PD to the handling editor.
